# HIV infection as a risk factor for vaginal dysbiosis, bacterial vaginosis, and candidosis in pregnancy: A matched case‐control study

**DOI:** 10.1111/birt.12526

**Published:** 2021-01-18

**Authors:** Philipp Foessleitner, Ljubomir Petricevic, Isabell Boerger, Irene Steiner, Herbert Kiss, Armin Rieger, Veronique Touzeau‐Roemer, Alex Farr

**Affiliations:** ^1^ Department of Obstetrics and Gynecology Division of Obstetrics and Feto‐maternal Medicine Medical University of Vienna Vienna Austria; ^2^ Center for Medical Statistics Informatics and Intelligent Systems (IMS) Medical University of Vienna Vienna Austria; ^3^ Department of Dermatology Medical University of Vienna Vienna Austria

**Keywords:** antenatal care, bacterial vaginosis, Candida, HIV/AIDS, pregnancy

## Abstract

**Background:**

This study aimed to evaluate the vaginal microbiota of HIV‐positive pregnant women relative to HIV‐negative controls, and to compare their risk of vaginal dysbiosis, bacterial vaginosis, and vulvovaginal candidosis (VVC).

**Methods:**

This is a nested matched case‐control study that analyzed data from women who received pregnancy care at our center from 2003 to 2014. Women routinely underwent screening for asymptomatic vaginal infections using phase microscopy on Gram‐stained smears. HIV‐positive women were assigned to the case group, and HIV‐negative women were assigned to the control group. Cases and controls were matched in a 1:4 ratio. Logistic regression was used to test whether HIV infection was associated with vaginal dysbiosis (Nugent score 4‐6), BV (Nugent score 7‐10), or VVC.

**Results:**

One hundred and twenty‐seven women were assigned to the case group, and 4290 were assigned to the control group (including 508 matched controls). Dysbiosis or BV was found in 29.9% of the cases and 17.6% of the controls. Women in the case group had increased risk of vaginal dysbiosis or BV (odds ratio [OR] 2.09, 95% confidence interval [CI], 1.30‐3.32, *P* = .002). The risk of VVC was also higher in the case group (OR 2.14, 95% CI, 1.22‐3.77, *P* = .008). The incidence of preterm birth did not differ significantly between the groups (cases: 8.7%; controls: 10%, *P* = .887).

**Conclusions:**

HIV‐positive women are at risk of vaginal dysbiosis, BV, and VVC during pregnancy. As imbalances of the vaginal microbiota can lead to preterm birth, screening and treatment of HIV‐positive pregnant women are warranted.

## 1 INTRODUCTION

The proportion of women among newly diagnosed HIV‐positive patients per year is approximately 25%, and 70% of these women are of reproductive age.^1^ HIV infection is not a limitation to conceive or to complete pregnancy, as the use of combination antiretroviral therapy (cART) is well tolerated and is associated with high life expectancy. The mother‐to‐child HIV transmission rate can effectively be reduced to less than 1% by following risk‐reduction measures, including antenatal and intrapartum antiretroviral treatment, abstaining from breastfeeding, and consideration of the viral load in the choice of the delivery mode.^2^ In a high‐resource country setting, HIV infection does not have an unfavorable effect on pregnancy and childbirth if it is treated with cART.^3^ Nevertheless, some studies have reported an increased rate of obstetric complications, for example, preterm birth (PTB), in HIV‐positive women.^4^, ^5^, ^6^

In 2001, a prospective cohort study reported that bacterial vaginosis (BV) has both a higher prevalence and an increased persistence in HIV‐positive, nonpregnant women, as compared to HIV‐negative women.^7^ Moreover, women with a CD4 cell count less than 200 cells/µL were shown to be more susceptible to BV than those with a CD4 cell count of more than 500 cells/µL. Furthermore, BV has been described as an independent risk factor for HIV infection,^8^ and a prospective study found higher rates of vulvovaginal candidosis (VVC) in HIV‐positive women than in HIV‐negative women.^9^

In pregnant women, vaginal dysbiosis, and BV in particular, represents causative factors in the multifactorial event of PTB.^10^, ^11^, ^12^, ^13^, ^14^ In addition to the association between BV and PTB,^15^, ^16^, ^17^ certain groups have an increased risk of vaginal infections, which underlines the importance of screening women for vaginal dysbiosis and for colonization by potentially harmful pathogens, in order to prevent them from ascending and causing preterm contractions, cervical insufficiency, and PTB.^11^, ^18^, ^19^ Among these risk groups are women receiving opioid‐maintenance therapy, who have an increased risk of vaginal infections.^20^ Screening pregnant women for infections even when they are asymptomatic has been reported to reduce the rate of PTB.^10^, ^11^ In HIV‐positive women, however, evaluation of the vaginal microbiota is not well established, despite the known importance as a result of the association between BV and PTB, and the high proportion of HIV‐positive women with low socio‐economic status, with substance use disorder, or on opioid‐maintenance therapy.^5^, ^20^

The present study sought to evaluate the vaginal microbiota of HIV‐positive pregnant women compared with that of negative controls, and their risk of vaginal dysbiosis, BV, and VVC. This information is important for midwives and obstetricians and may enable them to better identify women at risk who could benefit from antenatal screening for infection.

## 2 METHODS

### 2.1 Setting and study population

We conducted a retrospective analysis of data for all women with singleton pregnancies who received antenatal care at the Department of Obstetrics and Gynecology at the Medical University of Vienna, between January 1, 2003, and January 1, 2014. Our center is specialized in high‐risk pregnancy care, assisting in about 3000 births per year, including referrals from places throughout Central Eastern Europe. As part of our routine antenatal service, all asymptomatic women who register for a planned delivery at our department undergo infection screening during a prenatal consultation in early pregnancy. According to the official Austrian welfare program, further obstetric consultations are performed at predetermined time points in obstetric outpatient offices.^21^ As part of this pregnancy care, women routinely undergo HIV antibody screening using a laboratory‐based enzyme‐linked immunosorbent assay during the first trimester of pregnancy. Women with a positive HIV screening test result are transferred to our high‐risk obstetric center for multidisciplinary care, and undergo confirmatory HIV testing using immunoblot, and determination of HIV‐1 RNA levels by quantitative polymerase chain reaction. HIV‐1–positive women receive cART throughout their pregnancy, and their follow‐up includes routine determination of their viral load and CD4 cell count at predetermined time points. According to the national guidelines for the treatment of HIV‐positive women during pregnancy, the cART regimen consists of either a protease inhibitor (ie, lopinavir, atazanavir, darunavir, nelfinavir) or a non‐nucleoside reverse transcriptase inhibitor (eg, nevirapine), combined with two nucleoside transcriptase inhibitors (eg, zidovudine plus lamivudine or tenofovir plus emtricitabine).^22^ Antenatal care of HIV‐positive pregnant women is carried out in collaboration with the Department of Dermatology, involving a multidisciplinary team that consists of physicians, psychologists, nurses, and social workers.

### 2.2 Procedure

In our study, all women were asymptomatic and did not have visible signs of discharge or vaginal itching. As part of our routine protocol, vaginal smears were obtained by vaginal fluid collection with sterile swabs from the lateral vaginal wall and posterior fornix. Smears were Gram‐stained, and microscopy was performed by one of four biomedical laboratory assistants, who were trained and experienced in gynecological cytopathology, and laboratory certified according to DIN EN ISO 9001:2008. The cytopathologists were blinded to the medical history and HIV status of the women. The protocol involved classification of the vaginal microbiota as described by Nugent et al^23^ According to the scoring system,^23^ a score of 0‐3 was regarded as normal, 4‐6 as dysbiosis, and 7‐10 as BV. For the analyses, dysbiosis and BV were combined, as both conditions represent a disturbance of the normal microbiota.^24^ In addition, the presence or absence of *Candida* species (spp.) and/or *Trichomonas vaginalis (T vaginalis)* was assessed by microscopy. Women with BV received 2% clindamycin vaginal cream for 6 days (primary infection) or oral clindamycin (0.3 g) twice daily for 7 days (recurrent infection). Women with VVC received local clotrimazole (0.1 g) for 6 days, and those with trichomoniasis received local metronidazole (0.5 g) for 7 days.^25^ Antibiotic treatment was followed by vaginally applied *Lactobacillus* spp. for 6 days to rebuild the physiological microbiota and consequent follow‐up screening smears.^26^

### 2.3 Study groups

We conducted a matched‐group analysis to assess the effect of HIV infection on the observed outcome measures. Patients who underwent routine antenatal screening for asymptomatic infection at our department, with an available HIV screening test result, and aged 18‐45 years, were considered eligible. Cases and controls were matched in a 1:4 ratio according to the following parameters: parity (primipara versus [vs.] multipara), smoking status (smoker vs. nonsmoker), and maternal age (years). Each of the 127 cases was matched to four controls selected from the 4290 HIV‐negative women, which resulted in a total of 508 matched controls. Matching was performed using the R package matching version 4.9‐6 (R Development Core Team, R Foundation for Statistical Computing, Vienna, Austria).

### 2.4 Outcome measures

The vaginal microbiota at antenatal screening served as the primary outcome variable, which was recorded as either normal microbiota (±VVC) or dysbiosis/BV (±VVC). Secondary outcome variables included gestational age at birth (calculated according to measures on the sonogram at the first trimester screening using the equation by Hadlock et al^27^), neonatal birthweight, PTB (yes/no), VVC (yes/no), and trichomoniasis (yes/no). PTB was defined according to the World Health Organization definition^28^ as spontaneous or induced delivery at or less than 36 weeks plus 6 days of gestation. Stillbirth was defined as the term or preterm delivery of an infant who had died in utero and was born with an Apgar score of 0/0/0. Premature rupture of the membranes (PROM) was defined as the breakage of the amniotic sac before the onset of labor.^29^ Smoking status was evaluated by the number of cigarettes smoked *per day* at the time of the first visit to our department. Data were extracted from obstetric databases, patient charts, and microbiologic reports by using the PIA Fetal Database, version 5.6.16.917 (General Electric Company, GE Viewpoint, Munich, Germany).

### 2.5 Statistical analysis

Descriptive statistics were used to summarize demographic information. Continuous data were reported as the mean ± standard deviation, unless stated otherwise. Discrete data were reported as numbers (percentages). For the primary end point, an exact conditional logistic regression (SAS proc logistic) was performed based on the matching data set with the group (case vs. control) as an independent variable, and the matching group as a stratum variable. Odds ratios (ORs) with 95% confidence intervals (CIs) and the *P*‐value were used as a measure of risk. As results were dependent on the matched controls that were selected for the analyses, sensitivity analyses were conducted to evaluate the variability of the OR by applying the conditional regression model using ten additional randomly generated matching data sets. The minimum (min), maximum (max), and median of these ORs were calculated. The analyses of the secondary end points, VVC and PTB, were carried out analogously. A two‐sided *P*‐value < .05 was considered statistically significant. Statistical calculations were performed using R version 3.6.2 (http://www.r‐project.org/; R Development Core Team, R Foundation for Statistical Computing, Vienna, Austria) and SAS Statistical Analysis Software, version 9.4 (SAS Inc, Cary, NC, USA).

## 3 RESULTS

The data of 4517 consecutive pregnant with available screening smears were considered potentially eligible for the analysis. Of these women, those with incomplete or inconclusive data were excluded, leaving 4417 women, of whom 127 were identified as HIV positive. The 127 HIV‐positive women were assigned to the case group, and the remaining 4290 HIV‐negative women were assigned to the control group. Of the 4290 controls, 508 served as matched controls. Maternal characteristics of the overall 4417 women who were enrolled in the study are summarized in Table 1. Table 2 summarizes viral load, CD4 cell count, co‐infections, and prevalence of opioid‐maintenance therapy among the 127 women in the case group.

**TABLE 1:** Maternal characteristics of 4417 pregnant women in Austria, 2003‐2014

Variable	Cases (N = 127)	Matched controls (N = 508)	All controls (N = 4290)	All (N = 4417)
	N (%) Mean ± SD Median [Min–Max]	N (%) Mean ± SD Median [Min–Max]	N (%) Mean ± SD Median [Min–Max]	N (%) Mean ± SD Median [Min–Max]
Gravidity	2 [1‐8]	2 [1‐11]	2 [1‐22]	2 [1‐22]
Parity	2 [1‐8]	1 [1‐8]	2 [1‐10]	2 [1‐10]
Maternal age	30 ± 6	30 ± 6	31 ± 6	31 ± 6
Tertiary education				
Yes	0 (0.0)	35 (6.9)	345 (8.0)	345 (7.8)
No	127 (100.0)	473 (93.1)	3945 (92.0)	4072 (92.2)
Smoking				
Yes	36 (28.3)	144 (28.3)	749 (17.5)	785 (17.8)
No	91 (71.7)	364 (71.7)	3541 (82.5)	3632 (82.3)
Microbiota				
Normal	89 (70.1)	423 (83.3)	3533 (82.4)	3622 (82)
Dysbiosis	16 (12.6)	38 (7.5)	396 (9.2)	412 (9.3)
Bacterial vaginosis	22 (17.3)	47 (9.2)	361 (8.4)	383 (8.7)
VVC				
Yes	26 (20.5)	55 (10.8)	530 (12.4)	556 (12.6)
No	101 (79.5)	453 (89.2)	3760 (87.6)	3861 (87.4)
Trichomoniasis				
Yes	1 (0.8)	3 (0.6)	30 (0.7)	31 (0.7)
No	126 (99.2)	505 (99.4)	4260 (99.3)	4386 (99.3)

Abbreviations: max, maximum; min, minimum; SD, standard deviation; VVC, vulvovaginal candidosis.

**TABLE 2:** Maternal characteristics of 127 HIV‐positive pregnant women in Austria, 2003‐2014

Variable	N (%) Mean ± SD
Viral load^a^	
1st trimester	2.76 ± 1.41
2nd trimester	2.57 ± 1.24
3rd trimester	1.92 ± 0.79
CD4 cell count^a^	
1st trimester	439.18 ± 207.17
2nd trimester	442.49 ± 200.40
3rd trimester	494.25 ± 229.75
Opioid‐maintenance therapy	
Yes	18 (14.2)
No	109 (85.8)
Hepatitis B^b^	
Positive	6 (4.7)
Negative	121 (95.3)
Hepatitis C^b^	
Positive	22 (17.3)
Negative	105 (82.7)

Abbreviation: SD, standard deviation.

^a^ Viral load (HIV‐1 RNA levels) and CD4 cell count were available for 109 and 108 cases, respectively.

^b^ Serologic testing (surface antigen of the hepatitis B virus, anti‐hepatitis C virus), genotype, and viral load, as confirmed by polymerase chain reaction.

**FIGURE 1:**
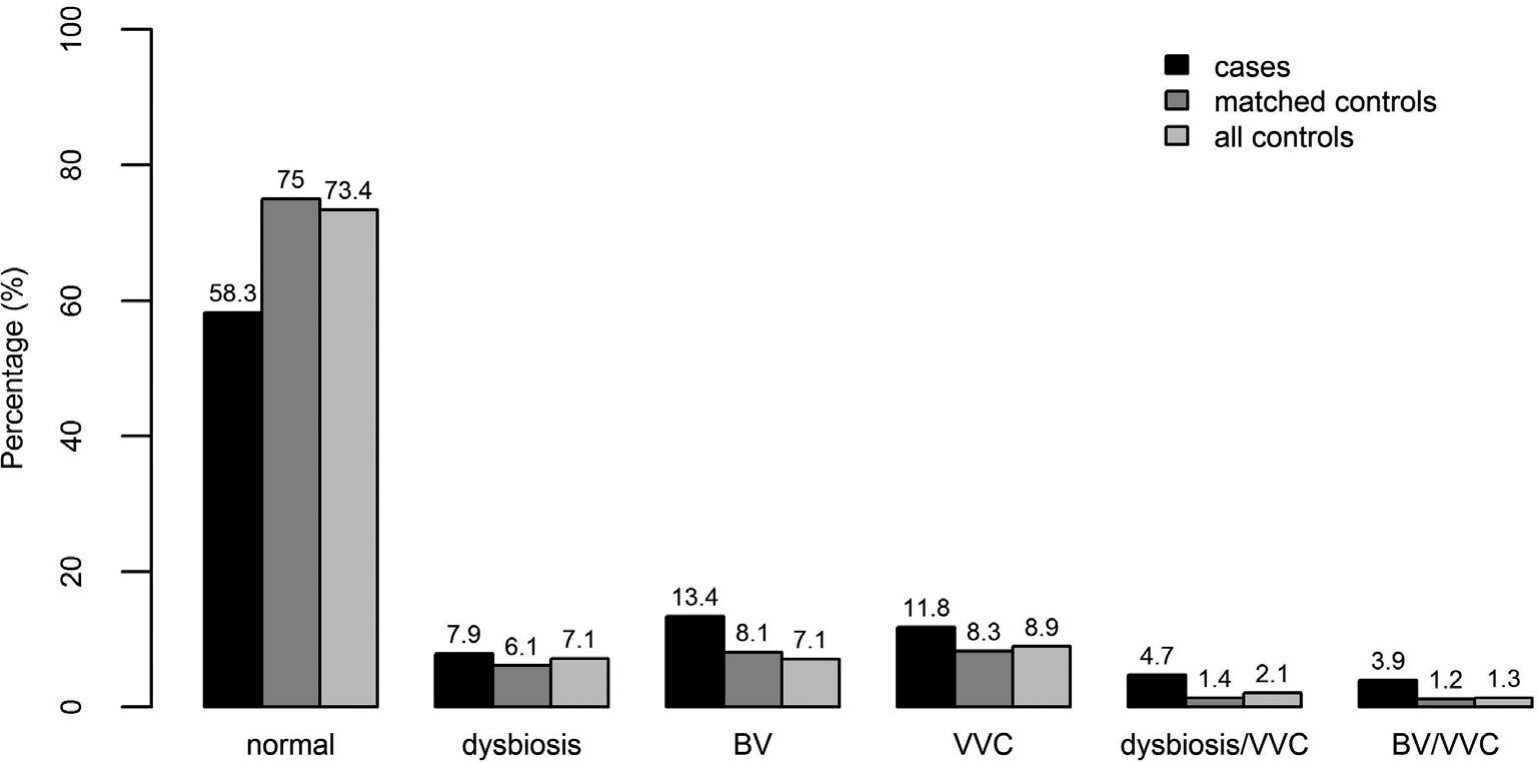
Vaginal microbiota of 4417 pregnant women in Austria, 2003‐2014 (BV, bacterial vaginosis; VVC, vulvovaginal candidosis)

The prevalence of vaginal dysbiosis or BV was 38 (29.9%) in the case group and 757 (17.6%) in the control group. Conditional logistic regression analysis showed a statistically significant difference in the occurrence of vaginal dysbiosis/BV (OR 2.09; 95% CI, 1.30‐3.32, *P* = .002). The prevalence of VVC was 26 (20.5%) in the case group and 530 (12.4%) in the control group. This difference between the groups was also statistically significant in the logistic regression analysis (OR 2.14; 95% CI, 1.22‐3.77, *P* = .008). Because of the small numbers, the difference in the prevalence of *T vaginalis* was not tested. The microbiota of the overall 4417 women enrolled in the study are shown in Figure 1.

The mean gestational age at birth was 38.7 ± 2.6 weeks and 38.8 ± 2.8 weeks in the case and control group, respectively. The mean birthweight was 2825 ± 598 g and 3242 ± 663 g in the case and control group, respectively. The incidence of PTB was 11 (8.7%) and 431 (10.0%) among women in the case and control group, respectively. This difference between the groups was not statistically significant in the logistic regression analysis (OR 0.89; 95% CI 0.40‐1.81, *P* = .887). Other obstetric outcomes of the 4417 women are shown in Table 3.

**TABLE 3:** Obstetric outcomes of 4417 pregnant women in Austria, 2003‐2014

Variable	Cases (N = 127)	Matched controls (N = 508)	All controls (N = 4290)	All (N = 4417)
	N (%) Mean ± SD Median [Min–Max]	N (%) Mean ± SD Median [Min–Max]	N (%) Mean ± SD Median [Min–Max]	N (%) Mean ± SD Median [Min–Max]
Gestational age at delivery	38.7 ± 2.6	38.7 ± 3.1	38.8 ± 2.8	38.8 ± 2.8
Neonatal birthweight	2825 ± 598	3190 ± 699	3242 ± 663	3230 ± 665
Birthweight percentile	31 ± 23	40 ± 27	44 ± 28	43 ± 28
Apgar score at 1 min < 7				
Yes	3 (2.5)	12 (2.4)	159 (3.7)	162 (3.7)
No	119 (97.5)	493 (97.6)	4101 (96.3)	4220 (96.3)
Apgar score at 5 min < 7				
Yes	1 (0.8)	7 (1.4)	69 (1.6)	70 (1.6)
No	121 (99.2)	498 (98.6)	4191 (98.4)	4312 (98.4)
Arterial umbilical cord pH	7.29 ± 0.07	7.27 ± 0.08	7.27 ± 0.08	7.27 ± 0.08
Preterm delivery				
Yes	11 (8.7)	49 (9.6)	431 (10.0)	442 (10.0)
No	116 (91.3)	459 (90.4)	3859 (90.0)	3975 (90.0)
PROM				
Yes	9 (7.1)	N/A	N/A	N/A
No	118 (92.9)	N/A	N/A	N/A
Mode of delivery				
Vaginal/instrumental	14 (11.0)	304 (59.8)	2505 (58.4)	2519 (57.0)
Cesarean	113 (89.0)	204 (40.2)	1785 (41.6)	1898 (43.0)

Abbreviations: max, maximum; min, minimum; N/A, data not available for controls; PROM, premature rupture of membranes; SD, standard deviation.

## 4 DISCUSSION

Imbalances in the vaginal ecosystem can lead to PTB,^10^, ^11^, ^12^, ^13^, ^14^ which is the main cause of neonatal morbidity and mortality worldwide. In our study, we found that HIV‐positive women had an increased risk of developing dysbiosis, BV, and VVC during pregnancy, when being compared to the controls.

Screening asymptomatic women for infection in early pregnancy has been shown to be effective.^10^, ^11^ However, as the body of literature is relatively limited, this should be confirmed by cohort studies in women with an increased risk of infection. Women who are HIV‐positive have an increased risk of obstetric complications, including PTB,^4^, ^5^, ^6^ which is a multifactorial event that is frequently caused by an overgrowth of anaerobic bacteria in the vaginal microbiota.^10^, ^11^, ^12^, ^13^, ^14^, ^30^ Of note, the risk of PTB was not increased in the cases of our study, which might be because infections were consequently treated and follow‐up smears were performed.

A previous study of nonpregnant women found a higher prevalence and an increased persistence of BV in HIV‐positive, nonpregnant women, compared with HIV‐negative women.^7^ This may also be the case for pregnant women. Taha et al^31^ tested pregnant African women for HIV and imbalances of their vaginal microbiota in late pregnancy, and found an association between the HIV positivity and BV. The authors hypothesized that the presence of BV may increase the risk of HIV acquisition during pregnancy. Our study, in contrast, found that BV in pregnancy was more likely to occur in women who had previously acquired an HIV infection.

Among nonpregnant women with HIV infection, the incidence and persistence of VVC is also increased.^9^ In general, immunocompromised women are at increased risk of fungal infections.^32^ The cases in our study had a greater than twofold increased risk of VVC compared with the controls, which may have been partially attributable to the cases being immunocompromised. Predisposing host factors such as HIV infection and other immunosuppressive diseases play a key role in the development of VVC.^33^, ^34^, ^35^ Although the use of cART is associated with a reduction in the incidence of severe opportunistic infections, with uneventful pregnancies and increased life expectancy of HIV‐positive women, opportunistic infections such as VVC remain prevalent in HIV‐positive individuals receiving cART.^34^, ^35^, ^36^ Previously conducted studies have shown that the increased vaginal colonization with fungi is caused by a loss of immunoprotective mechanisms.^37^, ^38^ In addition, proteinase activity, which plays an important role in the pathogenesis of VVC, is increased in HIV‐positive women and therefore renders them susceptible to VVC.^39^ We hypothesize that these factors may explain the increased incidence of VVC in the HIV‐positive group compared with those in the control group. This finding is of importance as recurrent VVC has been shown to increase the risk of PTB.^18^, ^40^ The pathogenic mechanism is unclear, but it may be associated with an inflammatory stimulus, leading to the release of cytokines and interleukins.^41^

In general, disruptions of the normal microbiota are thought to contribute to an increased susceptibility for PTB.^42^ A shift in the microbiota, not limited to a specific microorganism, is thought to be responsible for this phenomenon.^43^ An overgrowth of *Gardnerella vaginalis*, *Atopobium vaginae,* and other pathogens has been shown to increase the risk of late miscarriage and PTB.^16^ Previous studies have shown that BV,^10^, ^11^ vaginal dysbiosis,^44^ and recurrent VVC are associated with a higher risk of PTB.^18^ With regard to the risk of PTB, our results differ from some previous reports that HIV‐positive women have an increased incidence of PTB,^4^, ^5^, ^6^ as we found similar PTB rates in women with and without HIV infection. This may be a result of consequent treatment and follow‐up in case of vaginal infections. The high cesarean rate of 89% in HIV‐positive women in this study is because the national guidelines on pregnancy care recommend cesarean as the delivery mode of choice for HIV‐positive women.^22^

To the best of our knowledge, this is the first study to compare the vaginal microbiota of HIV‐positive and HIV‐negative pregnant women in the context of perinatal outcomes. Strengths of our study include the large control group and the use of matching. There are also some limitations; first, we did not evaluate all the maternal characteristics of interest such as co‐infections in healthy controls, nor the race/ethnicity of the women. However, data on race/ethnicity would likely not have affected our results, as the population at our center is relatively homogeneous. Second, we matched cases and controls for potentially confounding factors, but we were unable to adjust for poor obstetric history, which is a well‐established risk factor for PTB.^45^ It would have also been of interest to compare the PROM rate between the study and control group, but unfortunately, controls were derived from our large obstetric database, which, however, did not include PROM data. Finally, this cross‐sectional analysis cannot completely rule out that vaginal dysbiosis predated HIV, although this seems very unlikely. Indeed, our study findings should be confirmed by means of prospective studies.

In conclusion, this matched case‐control study demonstrates that HIV‐positive women have an increased risk of vaginal dysbiosis, BV, and VVC during pregnancy. We found no increased risk of PTB among HIV‐positive women, which may be a result of the consequent treatment and follow‐up in women with vaginal infections. However, as imbalances of the vaginal microbiota can lead to PTB, screening and treatment of asymptomatic infections is important for HIV‐positive pregnant women and should be implemented as part of their routine antenatal care.

## CONFLICT OF INTEREST

The authors declare none.

## ETHICAL APPROVAL

The ethics committee of the Medical University of Vienna approved this study (application number: 1335/2015). The study was performed in accordance with the Declaration of Helsinki and Good Scientific Practice guidelines, following the STROBE guidelines. Because of the retrospective design, the ethics committee issued a waiver of informed consent. All patient records were anonymized and de‐identified before the analyses.

## Data Availability

The data that support the findings of this study are available from the corresponding author upon reasonable request.
